# Outcomes of Hematopoietic Stem Cell Transplantation (HSCT) From Matched Related Donors in Pediatric Patients: Insights From a Single Center in the United Arab Emirates

**DOI:** 10.7759/cureus.89239

**Published:** 2025-08-02

**Authors:** Zainul Aabideen Kanakkande Kandy, Sagar Nivargi, Bina Chaudhary, Mohammed Ramzan, Fahed Abdullah, Fouzia Aboobacker, Charbel Khalil, Fulvio Porta, Lawrence Faulkner, Humaid Alshamsi

**Affiliations:** 1 Pediatric Hematology Oncology, Burjeel Medical City, Burjeel Cancer Institute, Abu Dhabi, ARE; 2 Pediatric Hematology Oncology and Bone Marrow Transplant (BMT), Burjeel Medical City, Burjeel Cancer Institute, Abu Dhabi, ARE; 3 Cell Therapy and Bone Marrow Transplant (BMT), Burjeel Medical City, Burjeel Cancer Institute, Abu Dhabi, ARE; 4 Bone Marrow Transplant (BMT), Burjeel Medical City, Burjeel Cancer Institute, Abu Dhabi, ARE; 5 Medical Oncology, Burjeel Medical City, Burjeel Cancer Institute, Abu Dhabi, ARE

**Keywords:** allogeneic hematopoietic stem cell transplantation, bmt, conditioning regimen, hematopoietic stem cell transplant, pediatrics & neonatology

## Abstract

Introduction

Allogeneic hematopoietic stem cell transplantation (HSCT) saves lives by treating a diverse range of pediatric conditions. Pediatric HSCT services began in the UAE in 2022. This study evaluates outcomes of the first 25 cases at Burjeel Medical City, Burjeel Cancer Institute, Abu Dhabi, following the establishment of the UAE's first pediatric bone marrow transplantation (BMT) unit.

Method

This retrospective study included children aged >30 days to <18 years who received HSCT from fully human leukocyte antigen (HLA)-matched related donors. Pre-transplant optimization and conditioning regimens were tailored to individual needs by a multidisciplinary team.

Results

The bone marrow served as the primary stem cell source in 96% of cases. Median engraftment times were 18 days for neutrophils and 15 days for platelets. Transplant-related mortality was 0%, with 4% graft failure and a 100% survival rate. Complications included bacterial sepsis (32%), acute graft-versus-host disease (GVHD) (20%), and chronic GVHD (12%).

Conclusion

Pediatric HSCT in the UAE has proven to be feasible and effective, offering a cost-efficient alternative to treatment abroad. It enhances access to advanced care within the region, marking a significant step forward in local pediatric healthcare and medical self-reliance.

## Introduction

Allogeneic hematopoietic stem cell transplantation (HSCT) is a potentially curative treatment for a wide range of hematological malignancies and non-malignant disorders in pediatric patients [1]. According to the updated 2022 classification of HSCT indications in children and adolescents, the scope of conditions treatable with HSCT includes hematological malignancies such as acute myeloid leukemia (AML), acute lymphoblastic leukemia (ALL), chronic myeloid leukemia (CML), myelodysplastic syndromes (MDS), and juvenile myelomonocytic leukemia (JMML) [2,3]. In addition, non-malignant disorders such as thalassemia major, sickle cell disease, inherited bone marrow failure syndromes, acquired aplastic anemia, inborn errors of immunity, primary hemophagocytic lymphohistiocytosis (HLH), mucopolysaccharidoses (MPS), lysosomal storage diseases, and metabolic bone diseases like osteopetrosis also benefit from HSCT [2].

HSCT in children is a complex and highly specialized procedure that necessitates comprehensive pre-transplant evaluation, extended hospital stays during the immediate post-transplant period, and intensive follow-up for at least one year [4,5]. Despite being widely practiced globally, pediatric HSCT services were not available in the United Arab Emirates (UAE) until recently, according to publicly available information. Prior to 2022, families seeking this life-saving treatment for their children had to travel abroad, often under challenging circumstances, particularly exacerbated during the COVID-19 pandemic. The pandemic highlighted the pressing need for local, advanced medical facilities, such as pediatric bone marrow transplantation (BMT) units, to minimize the burden on families and provide timely care.

In March 2022, the UAE achieved a significant milestone by performing the first pediatric allogeneic HSCT at Burjeel Medical City, Burjeel Cancer Institute, Abu Dhabi. This marked the establishment of the first pediatric BMT unit in the country, allowing children with life-threatening hematological and non-malignant disorders to receive cutting-edge treatment locally. However, setting up such a highly specialized unit poses numerous challenges, including infrastructure development, specialized personnel training, and implementing stringent post-transplant care protocols. This study aims to report early outcomes and challenges encountered during the establishment of the first pediatric HSCT program in the UAE. To the best of our knowledge, this is the first comprehensive report on pediatric allogeneic HSCT in the UAE. We discuss the experience of performing HSCT from a fully human leukocyte antigen (HLA)-matched related donor in 25 consecutive pediatric patients, aged between 30 days and 18 years, who underwent matched related donor HSCT for various indications from March 2022 to September 2024 and describe their outcomes.

## Materials and methods

This retrospective study included pediatric patients aged >30 days and <18 years who underwent allogeneic HSCT from an HLA-matched sibling or parent. Data were collected from the electronic medical records of the first 25 consecutive children who received HSCT from an HLA-matched related donor between March 2022 and September 2024 at Burjeel Medical City, Burjeel Cancer Institute, Abu Dhabi, UAE. Patient selection for HSCT was made according to institutional guidelines, which followed international standards. The study was approved by the Burjeel Institutional Review Board (BH/REC/061/24).

Pre-transplant preparation

Pre-transplant optimization was individualized based on the underlying condition. Patients with thalassemia major received adequate iron chelation, while those with sickle cell disease underwent exchange transfusions. For patients with primary immunodeficiency diseases (PID), infections were treated prior to transplantation, and patients with leukemia were induced into remission before proceeding to HSCT.

Conditioning regimen

The conditioning regimens were decided by a multidisciplinary team (MDT) based on the best available evidence and tailored to each patient's specific diagnosis (Table 1).

**Table 1:** Demographic details of the patients SCID: Severe Combined Immunodeficiency; HLH: Hemophagocytic Lymphohistiocytosis; ALL: Acute Lymphoblastic Leukemia; CSA: Cyclosporine A; MMF: Mycophenolate Mofetil; AFBC: Antibody-Free Bone Conditioning; GFTTh: Graft Facilitation with Thymoglobulin; TBI: Total Body Irradiation; GVHD: Graft Versus Host Disease

Patient Characteristics	N(%)
Age groups	
<1 year	2 (8%)
1–5 years	10 (40%)
5–10 years	9 (36%)
>10 years	4 (16 %)
Gender	
Female	16 (64%)
Male	9 (36%)
Diagnosis	
Thalassemia major	8 (32%)
Sickle cell disease	5 (20%)
Pure red cell aplasia	1 (4%)
Sideroblastic anemia	1 (4%)
Congenital amegakaryocytic thrombocytopenia	1 (4%)
SCID	2 (8%)
DOCK8 deficiency	1 (4%)
Chronic granulomatous disease	1 (4%)
HLH	3 (12%)
ALL	2 (8%)
Conditioning	
AFBC	15 (60%)
GFTTh	7 (28%)
GFTTh TBI	2 (8%)
GFT	1 (4%)
GFT	1 (4%)
GVHD prophylaxis	
CSA alone	1 (4%)
CSA + methotrexate	15 (60%)
CSA + MMF	9 (36%)

Graft-versus-host disease (GVHD) prophylaxis

GVHD prophylaxis was administered according to institutional protocols and included cyclosporine, methotrexate, and mycophenolate mofetil (MMF). Cyclosporine doses were adjusted to maintain a target trough level of 150-250 ng/mL. GVHD prophylaxis was gradually tapered beginning at day 100 for malignant disorders and day 180 for non-malignant disorders, if permitted (Table 1).

Bone marrow harvest and stem cell source

Bone marrow was the primary stem cell source, except in one case where peripheral blood stem cells (PBSC) were used. In 25 procedures, BM was harvested from the iliac crest under general anesthesia. The target cell dose was 6-8 × 10^8^ nucleated cells per kg of the recipient's body weight. The harvested bone marrow was collected in bags containing acid-citrate-dextrose (ACD).

Blood group mismatch management

For recipients with ABO major or bidirectional mismatch and anti-A or anti-B titers of 1:32 or higher, gradual transfusion of donor-type red cells was initiated. Starting with 5 mL of donor-specific blood on day -9 and slowly increasing to 10-15 mL on day -8, 40 mL on day -7, and 60 mL on day -6, if there is no reaction. On day -5, we test for antibody titer. We give another 50 mL of blood on day -1 if the titer is more than 1:3. In cases with persistently elevated titers, an additional infusion of donor-type blood was administered. Bone marrow grafts were infused without manipulation on the day of transplantation.

Post-transplant monitoring

After stem cell infusion, monitoring included blood tests for engraftment, organ function, blood product requirements, and viral reactivation. Chimerism analysis, which quantified the proportion of donor and recipient cells, was performed using short tandem repeat (STR) amplification techniques.

Supportive care

All patients were managed in a positive pressure, high-efficiency particulate air (HEPA)-filtered transplant unit. Prophylactic antimicrobials were initiated as per departmental guidelines. Discharge and follow-up care adhered to institutional protocols, including revaccination of all patients who had discontinued immunosuppression and showed no evidence of GVHD.

Definitions

Neutrophil engraftment was defined as three consecutive days with an absolute neutrophil count (ANC) >0.5 × 10^9^/L, while platelet engraftment was defined as an unsupported platelet count >20 × 10^9^/L for one week. Mixed chimerism was defined as donor cells constituting 5% to 95% of the recipient's hematopoietic system. Graft failure was categorized as primary (absence of donor hematopoietic reconstitution by day +28) or secondary (loss of donor cells after transient engraftment). Acute GVHD was graded using the Glucksberg criteria, and chronic GVHD was staged according to the Seattle criteria. Biopsies were obtained when clinically indicated to confirm the diagnosis of GVHD. Transplant-related mortality (TRM) was defined as any non-relapse death occurring within 100 days post-HSCT. Veno-occlusive disease (VOD) was diagnosed according to the European Society for Blood and Marrow Transplantation (EBMT) criteria. Transplant-associated thrombotic microangiopathy (TA-TMA) was diagnosed based on standard criteria.

Data collection and statistical analysis

Data collected included demographic information, indications for transplant, conditioning regimens (myeloablative versus reduced-intensity conditioning), GVHD prophylaxis, incidence of acute (grade III-IV) and chronic GVHD, bloodstream infections, sepsis, engraftment kinetics, relapse rates in malignant disorders, and TRM. Outcomes were measured by overall survival at 100 days post-HSCT, as well as GVHD- and relapse-free survival at the last follow-up. Descriptive statistics were used to summarize patient characteristics, with categorical variables expressed as percentages and continuous variables as medians with interquartile ranges (IQR).

Primary and secondary endpoints

The primary study endpoint was TRM. Secondary endpoints included the incidence of VOD, acute and chronic GVHD, mixed chimerism, TA-TMA, and other transplant-related complications. The cumulative incidence estimates were used to calculate rates of rejection, TRM, and GVHD.

## Results

The demographic characteristics of the 25 pediatric patients who underwent HSCT show that the majority of patients were between the ages of one and 10 years, with 10 children (40%) aged one to five years and nine children (36%) aged five to 10 years. Two patients (8%) were less than one year old, while five patients (20%) were older than 10 years. There was a slight female predominance in the cohort, with 16 female patients (64%) and nine male patients (36%).

The most common diagnosis among the patients was thalassemia major, accounting for eight patients (32%), followed by sickle cell disease in five patients (20%). Other conditions included HLH in three patients (12%), severe combined immunodeficiency (SCID) in two patients (8%), and ALL in two patients (8%). Less common diagnoses included pure red cell aplasia, sideroblastic anemia, congenital amegakaryocytic thrombocytopenia, DOCK8 deficiency, and chronic granulomatous disease, with each condition affecting one patient (4%).

Regarding conditioning regimens, 15 patients (60%) received a regimen of antibody-free bone conditioning (AFBC), while seven patients (28%) received graft facilitation with thymoglobulin (GFTTh). GFTTh with total body irradiation (TBI) was used in two patients (8%), and one patient (4%) received GFT. GVHD prophylaxis predominantly involved a combination of cyclosporine (CSA) and methotrexate, which was administered to 15 patients (60%). Nine patients (36%) received CSA with mycophenolate mofetil (MMF), and one patient (4%) received CSA alone (Table 1).

Characteristics of the donors

An overview of the donor characteristics for the 25 pediatric patients who underwent HSCT shows that the majority of donors were younger than 20 years, with 12 donors (48%) aged less than 10 years and nine donors (36%) aged between 10 and 20 years. Only five donors (20%) were older than 20 years. In terms of sex distribution, the majority of donors were female, accounting for 18 donors (72%), while eight donors (32%) were male. The donor-patient sex matching showed that 12 transplants (48%) involved female-to-female (F to F) donors and recipients. Female-to-male (F to M) transplants accounted for five cases (20%), male-to-male (M to M) transplants for four cases (16%), and male-to-female (M to F) transplants for five cases (20%). Regarding ABO blood group compatibility, 10 transplants (40%) were ABO compatible, while seven cases (28%) had a major ABO mismatch, and five cases (20%) had a minor ABO mismatch. Four cases (16%) involved bidirectional ABO mismatches. In terms of stem cell source, bone marrow was the predominant source, used in 25 transplants (96%), with only one case (4%) utilizing PBSC. This highlights bone marrow as the preferred graft source for pediatric patients in this cohort (Table 2).

**Table 2:** Characteristics of the donors (N=26) M: Male; F: Female; PBSC: Peripheral Blood Stem Cells

Characteristics of the Donor	N(%)
Donor age	
<10	12 (48%)
10–20	9 (36%)
>20	5 (20%)
Donor gender	
Male (M)	8 (32%)
Female (F)	18 (72%)
Donor-patient sex matching	
F to F	12 (48%)
F to M	5 (20%)
M to M	4 (16%)
M to F	5 (20%)
ABO blood group compatibility	
Compatible	10 (40%)
Major	7 (28%)
Minor	5 (20%)
Bidirectional	4 (16%)
Stem cell source	
Bone marrow	25 (96%)
PBSC	1 (4%)

Relationship between recipient and donor

The relationship between the recipients and their donors in the cohort shows that the most common donor-recipient relationship was sister-to-sister, accounting for 11 cases (44%), highlighting the predominance of female sibling donors. Sister-to-brother transplants were less frequent, occurring in three cases (12%), as were brother-to-brother transplants, which were also observed in three cases (12%). Brother-to-sister transplants occurred in five cases (20%), making it the second most common sibling relationship. Parental donations were less frequent, with mother-to-daughter transplants in one case (4%), mother-to-son transplants in two cases (8%), and father-to-son in one case (4%). These data reflect a higher occurrence of female donors, particularly sisters, in this cohort (Table 3).

**Table 3:** Relationship between recipient and donor (N=25)

Relationship b/w Recipient and Donor	N (%)
Sister to Sister	11 (44%)
Sister to Brother	3 (12%)
Brother to Brother	3 (12%)
Brother to Sister	5 (20%)
Mother to Daughter	1 (4%)
Mother to Son	2 (8%)
Father to Son	1 (4%)

Engraftment kinetics

The median time to neutrophil engraftment was 18 days, with a range of 14 to 22 days. Platelet engraftment occurred at a median of 15 days, with a wider range of 20 to 32 days. These data indicate that neutrophil recovery typically occurred earlier than platelet recovery, with variability in the platelet engraftment times among patients. The median times for both neutrophil and platelet engraftment fall within the expected range for pediatric HSCT (Table 4).

**Table 4:** Engraftment kinetics

Days to Engraftment	Median	Range
Neutrophils	18	14–22
Platelets	15	20–32

Graft failure, relapse, and mortality

The data provide an overview of key post-transplantation outcomes. Notably, there were no cases of relapse (0%), primary graft failure (0%), or TRM (0%) within the cohort. However, there was one case of secondary graft failure (4%). No deaths were recorded during the study period. These results demonstrate a favorable outcome for pediatric patients undergoing HSCT, with high survival rates and minimal serious complications post-transplant (Table 5).

**Table 5:** Graft failure, relapse, and mortality

Post-Transplantation Outcomes	N (%)
Relapse	0 (0%)
Primary graft failure	0 (0%)
Secondary graft failure	1 (4%)
Death	0 (0%)
Transplant-related mortality (TRM)	0 (0%)

Post-BMT complications

It outlines the post-transplant complications observed in the study cohort. Acute GVHD occurred in five patients (20%), with one patient (4%) experiencing severe grade III-IV GVHD. Chronic GVHD was seen in three patients (12%), with two having limited disease and two experiencing extensive GVHD. Infectious complications were relatively common, with bacterial sepsis reported in eight cases (32%), skin infections in six cases (24%), urinary tract infections (UTI) in four cases (16%), *Clostridioides difficile* infection in four cases (16%), and pneumonia in one patient (4%). Viral reactivations included cytomegalovirus (CMV) in three patients (12%) and BK virus in one patient (4%), while there were no cases of Epstein-Barr virus (EBV), adenovirus, or human herpesvirus 6 (HHV6) reactivations. Additionally, one patient (4%) developed a fungal infection. Importantly, no cases of VOD or TA-TMA were reported, highlighting the overall favorable post-transplant outcomes despite the presence of some infectious and GVHD-related complications (Table 6).

**Table 6:** Post-BMT complications GVHD: Graft-Versus-Host Disease; UTI: Urinary Tract Infection; CMV: Cytomegalovirus; EBV: Epstein-Barr Virus; HHV6: Human Herpesvirus 6; VOD: Veno-Occlusive Disease; TA-TMA: Transplant-Associated Thrombotic Microangiopathy

Post-Transplant Complications	N (%)
Acute GVHD	5 (20%)
None	0 (0%)
Grades II–IV	0 (0%)
Grades III–IV	1 (4%)
Chronic GVHD	3 (12%)
None	0 (0%)
Limited	2 (8%)
Extensive	2 (8%)
Bacterial sepsis	8 (32%)
C. difficile	4 (16%)
Skin infection	6 (24%)
UTI	4 (16%)
Pneumonia	1 (4%)
Rejection	1 (4%)
CMV reactivation	3 (4%)
EBV	0 (0%)
Adenovirus	0 (0%)
HHV6	0 (0%)
BK virus	1 (4%)
Fungal infection	1 (4%)
VOD	0 (0%)
TA-TMA	0 (0%)

## Discussion

HSCT is a life-saving treatment for a wide range of malignant and non-malignant diseases. The findings of our study demonstrate that pediatric allogeneic HSCT with fully HLA-matched related donors has yielded outstanding outcomes, with an overall survival rate of 100% in our first 25 consecutive cases. This study represents the first report of pediatric allogeneic HSCT outcomes from the UAE, highlighting the successful establishment of the country's first pediatric transplant center.

A fully HLA-matched donor has been consistently linked to better HSCT outcomes and reduced complication rates. In line with this, our approach as a new center was to initially focus on fully matched family donors for pediatric cases, which has likely contributed significantly to the absence of TRM [6-12]. Additional factors, such as targeted GVHD prophylaxis, optimized conditioning regimens, adequate total nucleated cell (TNC) dose, and the use of bone marrow as a stem cell source, were integral in achieving these outcomes. Importantly, the excellent quality of supportive care, availability of advanced antimicrobial agents, and vigilant CMV infection prevention and monitoring protocols have further bolstered patient outcomes.

Despite the heterogeneous diagnoses in our patient cohort, the HSCT outcomes were comparable to international standards, underscoring the effectiveness of our procedures and protocols even in a smaller cohort [7,8,10,13]. TRM remains a recognized challenge in pediatric HSCT, with various studies documenting TRM rates in children with different underlying conditions. However, the absence of TRM in our cohort attests to the robustness of our transplantation protocols and overall care approach. The absence of TRM in this study is a positive outcome and may be attributed to careful patient selection, pre-transplant conditioning, and close post-transplant monitoring [10,14,15].

An interesting observation in our study was that four of the 25 HLA-matched related donors were parents. This trend may be partially explained by the high prevalence of consanguineous marriages in our society, which increases the likelihood of a familial HLA match [10,13].

The demographic data revealed that thalassemia major remains a leading indication for HSCT in pediatric populations, especially in regions where the disease is more prevalent. Other significant indications included sickle cell disease (20%) and HLH (12%), which aligns with known HSCT indications in children [16]. In this cohort, 96% of the grafts were harvested from bone marrow, which remains the preferred stem cell source in pediatric HSCT due to lower rates of GVHD compared to PBSC.

The median time to neutrophil engraftment was 18 days, while platelet engraftment occurred at a median of 15 days. These engraftment times align with other studies involving pediatric HSCT patients, indicating effective early post-transplant recovery. The range of platelet engraftment, though wider (20-32 days), is also consistent with previous research, particularly in patients receiving bone marrow grafts, where platelet recovery can be slower compared to PBSC transplants [16].

GVHD remains one of the most serious complications following HSCT, with acute GVHD occurring in 20% of patients in this study. Only one patient (4%) experienced severe grade III-IV acute GVHD, which is consistent with the expected range reported in pediatric HSCT studies (20-40% for acute GVHD). Chronic GVHD was observed in 12% of patients, with a mix of limited and extensive disease, which also falls within the lower spectrum of previously reported rates (10-30%). The use of bone marrow as a source of stem cells might have contributed to the relatively low incidence of GVHD.

Infectious complications were more common, with bacterial sepsis reported in 32% of patients and skin infections in 24%. These findings are comparable to other pediatric HSCT studies, where infection rates tend to be higher due to the prolonged immunosuppression and neutropenia associated with the procedure. The study observed 16% of patients developing *Clostridium difficile* infections, which aligns with the known risk of gastrointestinal infections in post-HSCT patients. Additionally, CMV reactivation occurred in 12% of patients, reflecting the ongoing challenge of managing viral reactivations in immunocompromised patients post-transplant. Despite these infections, no fatalities were recorded, emphasizing the effectiveness of supportive care and early intervention protocols in this cohort.

Limitations

This study has several limitations that should be acknowledged. First, the small sample size (n=25) limits the statistical power and generalizability of the findings. Second, the absence of a comparator group prevents direct assessment of relative efficacy or outcomes against other treatment settings. Third, due to the limited cohort size and clinical heterogeneity, inferential statistical analyses and subgroup comparisons were not feasible and therefore not performed.

Fourth, the follow-up period was not uniform across patients, and as long-term outcome data were still being collected at the time of manuscript preparation, a reliable median follow-up duration could not be calculated. This variability limits the ability to assess the durability of outcomes.

Lastly, this is a single-center study, and results may reflect center-specific practices and resources, potentially limiting broader applicability.

## Conclusions

The study demonstrates significant progress in establishing a pediatric allogeneic HSCT unit at our center in the UAE. Our results, which align with international standards, confirm the feasibility of conducting HSCT locally. The outcomes from the first 25 patients are both encouraging and promising, highlighting the potential benefits for the pediatric population in the UAE. By offering this treatment domestically, we can reduce the economic and logistical burdens associated with traveling abroad for care while also ensuring continuity of care, an important factor for patients undergoing HSCT.
